# Alterations of bone mineral density, bone microarchitecture and strength in patients with ankylosing spondylitis: a cross-sectional study using high-resolution peripheral quantitative computerized tomography and finite element analysis

**DOI:** 10.1186/s13075-015-0873-1

**Published:** 2015-12-24

**Authors:** Nisha Nigil Haroon, Eva Szabo, Janet M. Raboud, Heather Mcdonald-Blumer, Lydia Fung, Robert G. Josse, Robert D. Inman, Angela M. Cheung

**Affiliations:** Department of Medicine, University of Toronto, Toronto, Canada; Toronto General Hospital, University Health Network, Eaton 7th Floor Rm 7EN221, 200 Elizabeth St., Toronto, ON M5G 2C4 Canada

**Keywords:** High-resolution peripheral quantitative computerized tomography, Bone mineral density, Ankylosing spondylitis, Osteoporosis, Bone microarchitecture

## Abstract

**Introduction:**

Ankylosing spondylitis (AS) is an inflammatory disease associated with new bone formation and an increased risk of osteoporosis and fractures. The negative effects of AS on bone microarchitecture and strength are unclear. Thus, we conducted an observational study to analyze the effect of AS on bone microarchitecture and strength.

**Methods:**

Patients with AS (n = 53) and non-AS subjects (n = 85) were recruited for the study. All subjects underwent clinical evaluation, DXA and high-resolution peripheral quantitative CT scans (HRpQCT).

**Results:**

The AS patients were aged 44 ± 12 (mean ± standard deviation) years and had a median disease duration of 17 (interquartile range: 7–27) years. They were found to have lower cortical, trabecular and total vBMD at the distal radius and tibia than non-AS subjects on multivariable regression analysis. Cortical parameters such as cortical thickness and porosity, and bone strength parameters such bone stiffness and stress as estimated by finite element analysis (FEA) in AS patients were significantly worse than that of-non-AS subjects. Among patients with AS, male sex, mSASSS greater than zero and HLA-B27 negative status were associated with worse bone microarchitecture.

**Conclusions:**

Patients with AS have worse bone mineral density, microarchitecture and strength when compared to non-AS subjects. More research is needed to understand the mechanisms underlying bone pathology in AS and to assess the effect of treatments such as TNF inhibitors on bone quality and fracture risk.

## Introduction

Ankylosing spondylitis (AS) is a spondyloarthropathic disorder that causes pain, fatigue, disability, morbidity, and mortality [1]. Patients with AS are at high risk of osteoporosis and vertebral fractures (VFs) [2]. In addition, non-VFs occur more frequently in AS patients [2, 3]. Patients with AS were found to have a 35 % increased risk of non-VFs in a large-scale study based on Danish health registries [3]. In 2014, a Spanish study reported a 19 % higher risk for non-VFs [2]. Fractures can cause severe pain, deformity, disability, poor quality of life, financial burden, and even mortality.

The etiology of fractures in AS is unclear. Peripheral fractures might be mediated by the increased risk of falls because of hyperkyphosis [4, 5]. Data regarding the association between low bone mineral density (BMD) and fractures in AS patients are inconsistent [6–10]. Both BMD and bone microarchitecture predict fracture risk [11, 12]. However, data on bone loss in patients with AS are based on studies using dual-energy X-ray absorptiometry (DXA). DXA-based BMD is two-dimensional and cannot distinguish between bone compartments. This is a limitation while studying inflammatory diseases causing differential involvement of the cortical and trabecular compartments. Other limitations of DXA in AS include false elevation of spine BMD by syndesmophytes and difficulty in patient positioning on the DXA table due to spinal immobility and coxitis [13, 14]. Further, some patients undergo hip replacement, making the hip scan difficult to interpret.

Earlier reports suggest that AS patients have low trabecular BMD in the spine [15]. HLA-B27 transgenic rats have abnormal trabecular structure in the spine and lower structural strength in the femur [16, 17]. Abnormal bone strength possibly occurs due to inflammation and high bone turnover [17]. These findings need to be confirmed using newer techniques such as high-resolution peripheral quantitative computerized tomography (HRpQCT) that provides compartment-specific (trabecular versus cortical) information. HRpQCT-based finite element analysis (FEA) provides knowledge about the biomechanical properties [18–20]. Our aim was to investigate the differences in volumetric bone mineral density (vBMD), bone microarchitecture, and strength in patients with severe AS and in non-AS subjects using HRpQCT and FEA. We also studied the influence of sex, modified Stoke Ankylosing Spondylitis Spine Score (mSASSS), and HLA-B27 on bone parameters.

## Methods

Patients were recruited from the spondylitis clinic located at Toronto Western Hospital, Canada. Our inclusion criteria were age >18 years and diagnosis of AS (modified New York criteria) [21]. All patients had been participating in a longitudinal study designed to assess the effect of tumor necrosis factor-alpha inhibitors (TNFi) on bone microarchitecture and hence had active disease (Bath Ankylosing Spondylitis Disease Activity Index (BASDAI) ≥4) [22]. Patients underwent all of the necessary laboratory tests, BMD, and HRpQCT just before starting TNFi. Eight patients declined participation, citing lack of time and significant pain. We excluded patients who had received TNFi and interleukin (IL)-12/23 inhibitors and pregnant women. Radiological severity of AS was documented by mSASSS (high mSASSS = mSASSS >0) [23]. The comparison group consisted of non-AS subjects who had participated in the Toronto HRpQCT substudy of the Canadian Multicenter Osteoporosis Study (CaMOS), a random population-based sample of noninstitutionalized individuals (aged ≥25 years) living within 50 km of Toronto [24].

### Assessment of areal BMD

Areal BMD was measured at the femoral neck, total hip, L1–L4 spine, and distal radius (Hologic, Bedford, MA, USA). Osteoporosis was defined using WHO criteria [25]. Premenopausal women and men aged <50 years were considered to have low bone mass if the *Z*-scores were ≤−2.0 [26].

### HRpQCT

HRpQCT was performed at the distal radius and tibia (XtremeCT; Scanco Medical, Bassersdorf, Switzerland). This scanner is equipped with a two-dimensional detector array and a 0.08 mm point-focus X-ray tube. The measurement settings were: X-ray tube potential of 60 kVp, current of 900 μA, and an image matrix size of 1536 × 1536. The limb positioning was secured inside a carbon-fiber shell. An anteroposterior scout view was acquired to determine the region of interest. A reference line was then placed at the end plate. We examined the areas of interest in 110 parallel two-dimensional slices. First slices were obtained at 9.5 and 22.5 mm proximal to the reference lines at the radius and tibia respectively. We separated the image slices into trabecular and cortical regions using a Gaussian filter and a threshold-based algorithm. We derived HRpQCT parameters using the standard morphological analysis [27]. The cortical bone was automatically segmented to measure geometry, density, and microstructure using a manufacturer-supplied algorithm [28]. The manufacturer’s phantom was scanned daily for quality control. We used HRpQCT images to estimate biomechanical parameters using FEA [18, 29, 30]. We used the voxel-conversion approach to generate linear, homogeneous finite-element meshes from the images. The boundary conditions simulated a uniaxial compression test. The nodes at the top and bottom surfaces were unconstrained and displacement was applied in the uniaxial testing direction that corresponded to 1 % strain. We applied a homogeneous tissue modulus of 6829 MPa and a Poisson’s ratio of 0.3.

### Statistical analysis

We summarized categorical and continuous variables as frequencies (percentages) and means (standard deviations) or medians (interquartile ranges) respectively. Two-sample *t* tests, Mann–Whitney U tests, or chi-square tests were used to compare intergroup differences. Multivariable linear regression analysis (adjusted for age and sex) was performed to study the effect of AS on HRpQCT parameters. We also examined the association between serum inflammatory markers and bone parameters using Pearson or Spearman correlation coefficients. The institutional research ethics board of the University Health Network, Toronto, Canada approved the study. All subjects gave written informed consent.

## Results

### Demographic parameters

The AS patients (*n* = 53) were mostly Caucasians (75 %) (Table 1). Two patients had VFs. Eight women were postmenopausal but none had received hormone therapy (HT).

**Table 1:** Clinical characteristics and areal BMD of AS patients and non-AS subjects

Variable	AS patients	Non-AS subjects
Number	53	85
Men (%)	29 (55)*	25 (29)
Caucasians	40 (75)*	76 (89)
Age (years)	44 ± 12*	61 ± 8
Weight (kg)	75 ± 3	76 ± 2
Height (cm)	167 ± 2	164 ± 1
BMI (kg/m^2^)	27 (22–32)	27 (25–31)
Fragility fractures (%)	5 (9)	6 (11)
Corticosteroid use (%)	3 (6)	5 (6)
Bisphosphonate use (%)	4 (9)	13 (15)
DMARDs (%)	7 (13)	0 (0)
Total daily calcium intake (mg)	745 (456–1020)*	1045 (471–1539)
Use of supplemental vitamin D (%)^a^	19 (36)	32 (38)
Hormone therapy (%)^b^	0 (0)*	16 (19)
Areal BMD^c^ (g/cm^2^)		
L1–L4	0.994 ± 0.179	0.988 ± 0.156
Total hip	0.904 ± 0.149	0.930 ± 0.123
Femoral neck	0.767 ± 0.142	0.756 ± 0.116
Distal 1/3 radius	0.740 ± 0.127	0.644 ± 0.170
Ultra distal radius	0.477 ± 0.078	0.418 ± 0.120
Low BMD/osteopenia (%)	18 (34)	37 (44)
Osteoporosis (%)	6 (11)	7 (8)

Data expressed as mean ± standard deviation, median (interquartile range) or number (% *N*) unless specified otherwise

^a^At least 1000 IU vitamin D

^b^Postmenopausal women receiving hormone replacement treatment

^c^Intergroup comparison of BMD was not performed between AS and non-AS subjects because of the differences in age and sex

*p <0.05

*AS* ankylosing spondylitis, *BMD* bone mineral density, *BMI* body mass index, *DMARD* disease-modifying antirheumatic drug

Patients with mSASSS >0 (*n* = 28) were significantly older and had longer disease duration than those with normal mSASSS (mSASSS = 0) (Table 2). Age, disease duration, sex distribution, mSASSS, BASDAI, and body mass index (BMI) did not differ between AS patients with or without HLA-B27 antigen (Table 2).

**Table 2:** Comparison of clinical parameters, HRpQCT, and BMD among AS patients based on sex, mSASSS, and HLA-B27

Variable	Total	Sex		mSASSS		HLA-B27	
	(*N* = 53)	Men	Women	mSASSS >0	mSASSS = 0	HLA-B27(+)	HLA-B27(−)
		(*n* = 29)	(*n* = 24)	(*n* = 28)	(*n* = 25)	(*n* = 36)	(*n* = 16)
Men (%)	29 (55)	–	–	20 (71)	9 (36)*	22 (61)	6 (38)
Caucasians	40 (75)	20 (69)	20 (83)	20 (71)	20 (80)	30 (83)	10 (63)
Age (years)	44 ± 12	45 ± 12	43 ± 11	48 ± 11*	39 ± 11	45 ± 12	40 ± 11
Disease duration (years)	17 (7–27)	21 (9–28)*	12 (4–20)	24 (9–32)*	12 (4–20)	20 (8–28)	11 (6–22)
BMI (kg/m^2^)	27 (22–32)	27 (25–32)	24 (21–32)	27 (24–30)	26 (22–32)	26 (22–29)	23 (27–36)
Current smoking (%)	20 (38)	13 (44)	7 (29)	12 (43)	8 (32)	14 (39)	5 (31)
Heavy alcohol use (%)^a^	2 (4)	2 (7)	0 (0)	1 (4)	1 (4)	1 (3)	1 (6)
Corticosteroid use (%)	3 (6)	1 (3)	2 (8)	0 (0)	2 (8)	0 (0)	2 (13)
Bisphosphonate use (%)	4 (9)	3 (16)	1 (5.3)	2 (7)	2 (8)	2 (5)	2 (11)
Use of DMARDs (%)	7 (13)	5 (17)	2 (8)	5 (18)	2 (8)	3 (8)	3 (19)
NSAID use (%)	47 (89)	26 (90)	21 (88)	25 (89)	22 (88)	29 (81)	10 (63)
IBD (%)	8 (18)	3 (10)	5 (13)	3 (10)	5 (20)	4 (11)	3 (19)
Psoriasis (%)	8 (18)	5 (17)	3 (13)	6 (20)	2 (8)	6 (17)	2 (13)
BASDAI	6.7 (4.8–7.9)	5.8 (4.3–7.0)*	7.5 (5.7–8.4)	6.5 (4.3–7.5)	6.8 (5.5–8.0)	6.6 (4.3–7.5)	7.5 (4.7–8.8)
mSASSS	2 (0–11)	8 (1–16)*	0 (0–2)	–	–	2 (0–15)	1 (0–7)
mSASSS > 0 (%)	28 (53)	20 (69)*	8 (33)	–	–	21 (58)	6 (38)
Fragility fractures (%)	5 (9)	4 (14)	1 (4)	4 (14)	1 (4)	4 (11)	2 (13)
Serum ESR (mm/hour)	21.9 ± 22.5	18 ± 19	25 ± 25	30.7 ± 26.9*	12.8 ± 10.9	21 ± 23	22 ± 20
Serum CRP (mg/l)	13.3 ± 16.7	13 ± 17	11 ± 15	19.6 ± 20.1*	6.1 ± 6.8	13 ± 18	12 ± 13
SAP (IU)	94 ± 41	88 ± 19	103 ± 60	106.8 ± 46.6*	80.7 ± 30.1	92 ± 41	99 ± 42

Data expressed as mean ± standard deviation, median (interquartile range) or number (% *N*) unless specified otherwise

^a^Heavy alcohol use: >14 drinks per week

*p <0.05, men vs. women

**p <0.05, high mSASSS vs. normal mSASSS

^†^ *p* <0.05, HLA-B27(+) vs. HLA-B27(−)

*AS* ankylosing spondylitis, *BASDAI* Bath Ankylosing Spondylitis Disease Activity Index, *BMD* bone mineral density, *BMI* body mass index, *CRP* C-reactive protein, *DMARD* disease-modifying antirheumatic drug, *ESR* erythrocyte sedimentation rate, *HRpQCT* high-resolution peripheral quantitative computerized tomography, *IBD* Inflammatory bowel disease, *mSASSS* modified Stoke Ankylosing Spondylitis Spine Score, *NSAID* nonsteroidal antiinflammatory drug, *SAP* Serum alkaline phosphatase

The non-AS patients (*n* = 85) were mostly Caucasians (89 %), had a mean age of 61 ± 8 years, and 71 % were women (Table 1). Five (6 %) and 13 non-AS subjects (15 %) used steroids and bisphosphonates respectively. Six (11 %) non-AS subjects had experienced fragility fractures and 16 (19 %) non-AS women had used HT. The total daily calcium intake was lower in AS patients than in non-AS patients (745 mg vs. 1045 mg, *p* <0.05). However, the use of supplemental vitamin D was similar between the two groups (36 % vs. 38 %).

### Abnormalities in vBMD, bone structure, and strength in AS patients in comparison with non-AS subjects

Multivariable regression analysis adjusted for differences in age and sex suggested that patients with AS (*n* = 53) had lower cortical and total vBMD at the distal radius and tibia than non-AS subjects (*n* = 85) (Table 3). Cortical thickness was reduced and cortical porosity increased in AS patients. Trabecular microarchitecture was not different between the two groups. FEA parameters at the radius (bone stiffness and stress) were negatively affected in AS patients (Table 3). Similar changes were noted at the distal tibia, and the beta coefficient showed a trend towards being statistically significant (Table 3).

**Table 3:** Multivariable linear regression analysis assessing AS as an independent predictor of BMD, and bone microarchitecture

HRpQCT	Radius				Tibia			
	β^a^	95 % CI	*p* value	Adj. *R* ^2^	β^a^	95 % CI	*p* value	Adj. *R* ^2^
Trabecular vBMD	−15.1	−33.3 to –3.2	0.105	0.184	−17.2	−35.3 to –1.5	0.071	0.094
Cortical vBMD	−34.3	−62.8 to –7.9	0.011	0.160	−28.15	−55.1 to –5.4	0.018	0.096
Total vBMD	−41.6	−71.0 to –12.1	0.006	0.087	−28.13	−51.7 to 4.5	0.020	0.256
Trabecular number	–0.088	–0.244 to –0.068	0.268	0.070	–0.065	−0.343 to 0.113	0.471	0.063
Trabecular thickness	–0.004	–0.008 to 0.001	0.087	0.217	–0.004	–0.010 to 0.003	0.260	0.001
Trabecular separation	0.052	0.011–0.115	0.105	0.072	0.060	–0.017 to 0.137	0.128	0.063
BV/TV (%)	–0.013	–0.028 to –0.003	0.106	0.184	–0.014	–0.030 to –0.001	0.070	0.094
Cortical thickness	–0.101	–0.163 to –0.039	0.002	0.202	–0.060	–0.116 to 0.004	0.035	0.381
Cortical porosity	0.006	0.001–0.010	0.016	0.326	0.013	0.002–0.023	0.016	0.323
Stiffness	–0.226	–0.384 to –0.069	0.005	0.055	–0.160	–0.333 to 0.013	0.069	0.081
Stress	−5.7	−9.61 to –1.7	0.005	0.055	−4.0	−8.3 to 0.24	0.064	0.082

^a^Beta coefficient represents the difference between patients with AS (*n* = 53) and subjects without AS (*n* = 85), and subjects without AS are the reference category. The model was adjusted for differences in age and sex

*Adj.* adjusted, *AS* ankylosing spondylitis, *BMD* bone mineral density, *BV/TV* bone volume/trabecular volume, *CI* confidence interval, *HRpQCT* high-resolution peripheral quantitative computerized tomography, *vBMD* volumetric bone mineral density

### Comparison of HRpQCT parameters in AS patients with mSASSS >0 vs. mSASSS = 0

Intergroup comparisons performed using two-sample *t* tests showed that AS patients who had mSASSS >0 had worse bone microarchitecture than those who had normal mSASSS (mSASSS = 0) (Table 4). Specifically, the high-mSASSS group (*n* = 28) had lower cortical vBMD, greater cortical porosity, and lower cortical thickness at both sites. Bone stiffness and stress also tended to be lower although these results did not reach statistical significance. Trabecular parameters did not differ between the groups. On multivariable regression analysis, the negative effect of high mSASSS tended to be statistically significant for cortical vBMD (radius) and cortical porosity (tibia), after adjusting for age and disease duration (β = −27.1, 95 % confidence interval (CI): −56.0 to 1. 4, *p* = 0.05; and β = .21, 95 % CI: −0.01 to 0.3, *p* = 0.09 respectively).

**Table 4:** Comparison of HRpQCT and BMD among patients with AS based on sex, mSASSS, and HLA-B27

Variable	Total	Sex		mSASSS		HLA-B27	
	(*N* = 53)	Men	Women	mSASSS > 0	mSASSS = 0	HLA-B27(+)	HLA-B27(−)
		(*n* = 29)	(*n* = 24)	(*n* = 28)	(*n* = 25)	(*n* = 36)	(*n* = 16)
HRpQCT at radius							
Trabecular vBMD (mg/cm^3^)	165.3 ± 45.7	186.1 ± 39.6*	140.0 ± 40.0	168.8 ± 54.4	161.3 ± 34.2	175.7 ± 39.9	142.8 ± 52.3^†^
Cortical vBMD (mg/cm^3^)	854.7 ± 66.2	826.8 ± 51.8*	864.0 ± 55.2	821.6 ± 57.8**	868.4 ± 43.1	845.3 ± 59.2	834.0 ± 90.9
Total vBMD (mg/cm^3^)	315.4 ± 64.4	322.5 ± 62.8	306.8 ± 66.7	303.42 ± 74.28	328.88 ± 49.49	324.0 ± 57.5	300.3 ± 77.4
Trabecular number (/mm^3^)	2.02 ± 0.34	2.13 ± 0.24*	1.86 ± 0.43	1.99 ± 0.44	2.04 ± 0.25	2.07 ± 0.29	1.89 ± 0.48
Trabecular thickness (mm)	0.068 ± 0.012	0.073 ± 0.012*	0.062 ± 0.010	0.069 ± 0.013	0.066 ± 0.011	0.071 ± 0.01	0.061 ± 0.01^†^
Trabecular separation (mm)	0.457 ± 0.185	0.402 ± 0.061*	0.523 ± 0.254	0.479 ± 0.246	0.432 ± 0.068	0.423 ± 0.08	0.535 ± 0.31^†^
BV/TV (%)	0.138 ± 0.038	0.155 ± 0.033*	0.117 ± 0.033	0.141 ± 0.045	0.134 ± 0.029	0.147 ± 0.03	0.119 ± 0.40^†^
Cortical thickness (mm)	0.765 ± 0.153	0.748 ± 0.148	0.786 ± 0.159	0.715 ± 0.159**	0.821 ± 0.126	0.770 ± 0.165	0.760 ± 0.160
Cortical porosity (%)	0.020 (0.010–0.025)	0.022 (0.018–0.026)*	0.010 (0.008–0.020)*	0.021 (0.019–0.027) **	0.012 (0.009–0.018)	0.020 (0.016–0.024)	0.016 (0.009–0.023)
Stiffness (N/mm)	1.4 ± 0.4	1.4 ± 0.4	1.3 ± 0.4	1.3 ± 0.04	1.5 ± 0.4	1.4 ± 0.4	1.3 ± 0.5
Stress (MPa)	27.7 ± 9.4	28.72 ± 9.35	26.38 ± 9.57	26.0 ± 9.9	29.5 ± 8.7	28.8 ± 8.9	25.8 ± 10.7
HRpQCT at tibia							
Trabecular vBMD (mg/cm^3^)	159.9 ± 43.7	176.6 ± 38.5*	139.8 ± 41.9	162.8 ± 44.8	157.4 ± 43.5	170.9 ± 40.7	136.9 ± 43.4^†^
Total vBMD (mg/cm^3^)	296.4 ± 55.5	302.9 ± 46.0	288.5 ± 65.1	285.1 ± 57.2	308.2 ± 52.1	309.4 ± 39.3	272.5 ± 74.8^†^
Cortical vBMD (mg/cm^3^)	843.7 ± 56.1	848.4 ± 43.4	862.2 ± 86.7	835.2 ± 79.3**	876.9 ± 39.1	865.4 ± 50.8	834.0 ± 91.0
Trabecular number (per mm^3^)	1.89 ± 0.45	2.05 ± 0.29*	1.70 ± 0.533	1.95 ± 0.42	1.84 ± 0.47	1.96 ± 0.42	1.71 ± 0.49
Trabecular thickness (mm)	0.07 ± 0.01	0.070 ± 0.014	0.071 ± 0.015	0.070 ± 0.013	0.073 ± 0.015	0.073 ± 0.014	0.070 ± 0.011
Trabecular separation (mm)	0.50 ± 0.23	0.43 ± 0.07*	0.60 ± 0.31	0.47 ± 0.13	0.53 ± 0.29	0.46 ± 0.13	0.60 ± 0.35^†^
BV/TV (%)	0.133 ± 0.036	0.147 ± 0.032*	0.117 ± 0.035	0.131 ± 0.037	0.136 ± 0.036	0.143 ± 0.034	0.114 ± 0.036^†^
Cortical thickness (mm)	0.744 ± 0.180	0.695 ± 0.137*	0.804 ± 0.208	0.691 ± 0.182**	0.804 ± 0.161	0.766 ± 0.185	0.705 ± 0.169
Cortical porosity (%)	0.049 (0.035–0.067)	0.061 (.044–0.075)**	0.039 (0.029–0.056)**	0.062 (0.045–0.091)**	0.040 (0.033–0.056)	0.047 (0.032–0.070)	0.056 (0.035–0.071)
Stiffness (N/mm)	1.51 ± 0.42	1. 6 ± 0.3	1.5 ± 0.4	1.6 ± 0.3	1.5 ± 0.4	1.6 ± 0.3	1.4 ± 0.4^†^
Stress (MPa)	31.6 ± 9.1	32.7 ± 8.0	31.1 ± 9.5	30.6 ± 8.7	33.5 ± 8.5	33.8 ± 7.2	28.8 ± 10.4^†^
L1–L4 BMD	0.994 ± 0.179	1.018 ± 0.163	0.964 ± 0.198	1.001 ± 0.214	0.986 ± 0.138	1.024 ± 0.182	0.944 ± 0.161
Total hip BMD	0.904 ± 0.149	0.943 ± 0.125*	0.858 ± 0.165	0.887 ± 0.158	0.921 ± 0.140	0.925 ± 0.123	0.857 ± 0.197
Femoral neck BMD	0.767 ± 0.142	0.795 ± 0.153	0.733 ± 0.124	0.735 ± 0.125	0.798 ± 0.153	0.786 ± 0.130	0.727 ± 0.169
Distal 1/3 radius BMD	0.740 ± 0.127	0.802 ± 0.067*	0.659 ± 0.142	0.771 ± 0.080	0.708 ± 0.156	0.764 ± 0.088	0.679 ± 0.185^†^
Ultradistal radius BMD	0.477 ± 0.078	0.505 ± 0.079*	0.441 ± 0.061	0.481 ± 0.086	0.472 ± 0.069	0.488 ± 0.084	0.453 ± 0.059

Data expressed as mean ± standard deviation, median (interquartile range) or number (% *N*) unless specified otherwise

*p <0.05, men vs. women

**p <0.05, high mSASSS (mSASSS >0) vs. normal mSASSS

^†^ *p* <0.05, HLA-B27(+) vs. HLA-B27(−)

*AS* ankylosing spondylitis, *BMD* bone mineral density, *BV/TV* bone volume/trabecular volume, *HRpQCT* high-resolution peripheral quantitative computerized tomography, *mSASSS*, modified Stoke Ankylosing Spondylitis Spine Score, *vBMD* volumetric bone mineral density

### Sex differences in HRpQCT parameters in AS patients

Intergroup comparisons performed using two-sample *t* tests showed that women had worse trabecular microarchitecture than men but cortical bone abnormalities were more predominant in men (Table 4). Trabecular vBMD and bone volume/total volume (BV/TV) were lower in women. Women had fewer, thinner, and more widely spaced trabeculae than men. However, men had lower cortical vBMD at the radius, cortical thickness at the tibia, and greater cortical porosity at both sites. The differences in HRpQCT parameters persisted even after excluding postmenopausal women. No differences existed in bone strength between men and women.

### Comparison of HRpQCT characters in AS patients with or without HLA-B27

AS patients without HLA-B27 antigen (*n* = 16) were found to have lower trabecular vBMD in comparison with AS patients with HLA-B27 (Table 4). In addition, BV/TV and trabecular separation and FEA parameters at the tibia were abnormal in HLA-B27(−) patients. Intergroup comparisons were performed using two-sample *t* tests.

Regression analysis in patients with AS alone did not suggest that parameters such as the BASDAI and smoking had an influence on bone microarchitecture and strength.

### Areal BMD abnormalities in AS patients

The prevalence of osteoporosis was 10 % (3/29) and 13 % (3/24) in men and women with AS respectively. Approximately 31 % of men and 38 % of women had osteopenia or low bone mass and 6/8 postmenopausal women had either osteoporosis or osteopenia. Although men with AS had more cortical bone abnormalities, BMD at the total hip and radius was higher in men than in women (Table 4). Despite women having more trabecular abnormalities at the distal sites on HRpQCT, lumbar spine BMD was similar between men and women. Areal BMD at most sites did not differ based on HLA-B27 status or mSASSS (Table 4). On multivariable regression analysis, when compared with non-AS subjects (*n* = 85), patients with AS (*n* = 53) had lower BMD at both the total hip and femoral neck even after adjusting for age and sex (Table 5).

**Table 5:** Multivariable linear regression analysis assessing AS as an independent predictor of areal BMD

	β^a^	95 % confidence interval	*p* value	Adjusted *R* ^2^
L1–L4 spine	–0.02	–0.10 to 0.06	0.656	0.097
Total hip	–0.11	–0.17 to –0.05	0.000	0.223
Femoral neck	–0.09	–0.15 to –0.37	0.001	0.242
Distal 1/3 radius	0.01	–0.03 to 0.04	0.926	0.316
Ultradistal radius	–0.02	–0.05 to 0.02	0.421	0.316

^a^Multivariable models adjusted for age and sex

*AS* ankylosing spondylitis, *BMD* bone mineral density

### Correlation between erythrocyte sedimentation rate, C-reactive protein, and HRpQCT parameters

We observed significant negative correlations between erythrocyte sedimentation rate (ESR) and tibial vBMD. The correlation coefficients between ESR and vBMD at the distal tibia were −0.351(*p* <0. 05), −0.382 (*p* <0.001), and –0.356 (*p* <0.05) for trabecular, cortical, and total vBMD respectively. Cortical thickness (*r* = −0.297, *p* <0.05) and BV/TV (*r* = −0.351, *p* <0.001) at the distal tibia also correlated negatively with ESR. Statistically significant inverse correlation was also noted between bone stiffness (*r* = −0.293, *p* <0.05) and stress (*r* = −0.378, *p* <0.001) and serum ESR. Conversely, serum C-reactive protein (CRP) was not correlated with HRpQCT parameters. No correlation existed between areal BMD and ESR or CRP.

## Discussion

Our results suggest that AS patients have lower cortical vBMD, lower cortical thickness, higher cortical porosity, and lower bone stiffness and stress when compared with non-AS controls. These abnormalities might partly explain the cause for fractures in AS. Areal BMD at the lumbar spine and distal radius did not differ between AS and non-AS subjects despite differences in bone microarchitecture.

HRpQCT-based measures of bone quality can predict fractures independent of BMD [29–31]. We found that bone stiffness and stress (as estimated by FEA) were significantly lower at the distal radius in AS patients. These abnormalities were not as pronounced at the distal tibia, probably because of the protective effect of skeletal loading.

Our results confirm the findings of a recent cross-sectional study in which cortical vBMD at the distal radius and trabecular vBMD at the distal tibia were reduced in AS patients [32]. Similar to our study, the authors noted that cortical porosity was higher in AS patients. It is now thought that VFs are associated with lower vBMD and cortical thinning [32]. However, certain methodological differences exist between our study and the study by Klingberg et al. The main differences are that only men were studied by Klingberg et al., and cases and controls were not ethnically or geographically matched introducing bias from unmeasured confounders. In addition, the description of the abnormalities of bone microstructure in patients who had VFs were based on a small sample (*n* = 8) and only morphometric VFs belonging to Genant grade 1 category (12/14 VFs) were studied [32]. Genant grade 1 fractures are not always caused by osteoporosis and may not predict future VFs. Although disease-modifying antirheumatic drugs (DMARDs; methotrexate and sulfasalazine) have a neutral effect on areal BMD, use of TNFi is associated with improvement in spine and hip BMD in AS patients [33–35]. However, Klingberg et al. did not account for the influence of methotrexate, sulfasalazine, or TNFi despite the fact that a significant number of patients had received such medications. Conversely, in our study, the use of bisphosphonates, DMARDs, and steroids was negligible and no patient had received TNFi.

HLA-B27 might have a role in mediating bone loss in AS. HLA-B27 transgenic rats experience bone loss due to increased osteoclastogenesis and bone resorption [17]. However, data on the effect of HLA-B27 on bone loss in AS patients are inconsistent. In our study, the HLA-B27(−) AS patients had worse trabecular parameters, bone stiffness, and stress than the HLA-B27(+) patients. This supports the results of a previous study [36] that reported a higher prevalence of clinical VFs in HLA-B27(−) patients (9.9 vs. 4.8 %) than HLA-B27(+) patients. In contrast, another study found that AS patients with low BMD or VFs were more likely to be HLA-B27(+), but the difference was not statistically significant [37].

We found important sex differences in bone microarchitecture and strength in AS. Although men had higher areal BMD at the total hip and distal radius than women, the cortical bone vBMD and microarchitecture were worse in men. Conversely, trabecular parameters were worse in women. Men with AS had greater cortical porosity despite the fact that cortical porosity is worse in healthy women than men during young adulthood [38]. This is possibly due to men having more severe disease than women. Factors such as menopause and systemic inflammation are probably responsible for the abnormal trabecular microarchitecture in AS women [38, 39], which is worse than in men. We observed no sex differences in bone stiffness in AS patients. However, it is established that in young healthy adults, bone stiffness is lower in women than in men [40]. Longstanding disease and higher mSASSS in men may have caused a stronger insult on FEA parameters in men masking the sex differences. Our results thus suggest that sex differences in bone microarchitecture and strength are attenuated by AS.

We found that patients with mSASSS >0 tended to have poor cortical bone microarchitecture. These results were partly influenced by differences in age and disease duration, however, and need to be studied further. Abnormal cortical microarchitecture in those with mSASSS >0 may indicate that systemic bone loss occurs in parallel with radiological progression of AS. In the study by Klingberg et al. [32], the mSASSS correlated with abnormal cortical porosity and cortical vBMD.

Our results showing that AS patients have abnormal cortical microarchitecture are important given that the microstructure of cortical bone is a key determinant of bone fragility [31, 41–43]. Cortical microarchitecture was negatively affected at both the distal radius and tibia, suggesting that mechanical loading may not ameliorate the catabolic actions of inflammation on cortical bone. Age-related loss of cortical bone does not begin until middle age or late life in women [44, 45]. Patients with AS had poor cortical bone structure despite being young and it is likely that AS exaggerates loss of cortical bone. Abnormal cortical bone parameters in AS are probably due to systemic inflammation, vitamin D deficiency, secondary hyperparathyroidism, abnormal skeletal loading due to abnormal gait and posture, low peak bone mass, low BMI, low lean mass, hypogonadism, malabsorption, inflammatory bowel disease (IBD), and trabecularization of cortical bone [46–50].

Systemic inflammation plays a key role in causing bone loss, as suggested by the negative correlation between ESR and HRpQCT parameters. AS is characterized by ossification of the enthuses and formation of osteophytes but paradoxically there is loss of trabecular bone adjacent to the sites of inflammation. Trabecular and cortical compartments appear to have different reactions to inflammation. Although a high BASDAI indicates severe AS, we found that it was not associated with poor bone microarchitecture. This is in concordance with past reports that the BASDAI may not differentiate AS patients with low BMD [34, 35]. Subjective measures such as the BASDAI may not reflect longstanding inflammation. Furthermore, all our patients had a high BASDAI and the lack of variability in our population may have influenced our results.

Our study has both limitations and strengths. The main limitations include the cross-sectional design, small sample size, and lack of generalizability in non-Caucasian patients. Despite the small size of our study, it is the largest study of bone microarchitecture in AS patients (including both men and women), to our knowledge. We may have missed some differences in bone microarchitecture in the secondary analyses due to lack of power. All patients had active disease and hence the results may only reflect abnormalities related to severe inflammation. Next, 18 % of patients had IBD and this may have affected our results. Also, we did not study testosterone levels and bone turnover. Another limitation is that we did not have data on smoking, alcoholism, and physical activity in the non-AS group. Finally, we could not study the discriminative ability of HRpQCT to identify patients with high fracture risk since the number of patients with fractures was small. Our study has several strengths including the assessment of bone strength and stiffness in AS patients for the first time. This is only the second study to provide detailed information of bone microarchitecture in patients with AS. Our results may guide future research on the prediction and management of fragility fractures in AS patients. We were able to generate important findings that both trabecular and cortical compartments are affected in AS. This information can be used to design future studies aimed at assessing changes in bone strength in response to NSAIDs or TNFi. Finally, the potential confounding effect of TNFi was eliminated by excluding subjects who had been on treatment with TNFi, whereas previous studies were unable to do so [32, 33].

## Conclusions

We conclude that vBMD, bone microstructure, and strength are abnormal in AS patients with severe disease. Sex, mSASSS, and HLA-B27 are determinants of bone microarchitecture in AS. Future research should investigate whether abnormal bone microarchitecture and strength are predictive of fractures in AS. The effect of TNFi on bone strength also needs to be studied.

## Abbreviations

AS: Ankylosing spondylitis
BASDAI: Bath Ankylosing Spondylitis Disease Activity Index
BMD: Bone mineral density
BMI: Body mass index
BV/TV: Bone volume/total volume
CaMOS: Canadian Multicenter Osteoporosis Study
CI: Confidence interval
CRP: C-reactive protein
DMARD: Disease-modifying antirheumatic drug
DXA: Dual-energy X-ray absorptiometry
ESR: Erythrocyte sedimentation rate
FEA: Finite element analysis
HT: Hormone therapy
IBD: Inflammatory bowel disease
HRpQCT: High-resolution peripheral quantitative computerized tomography
IL: Interleukin
mSASSS: Modified Stoke Ankylosing Spondylitis Spine Score
TNFi: Tumor necrosis factor inhibitors
vBMD: volumetric bone mineral density
VF: Vertebral fracture
